# Transforming Agri-food waste: Innovative pathways toward a zero-waste circular economy^☆^

**DOI:** 10.1016/j.fochx.2025.102604

**Published:** 2025-05-30

**Authors:** Arun Kumar Pandey, Sheetal Thakur, Rahul Mehra, Raj Sukhwinder Singh Kaler, Maman Paul, Arun Kumar

**Affiliations:** a Amity Institute of Biotechnology, Amity University, Rajasthan, Jaipur 303002, India; b University Centre for Research & Development, Department of Biotechnology, Chandigarh University, Gharuan, Mohali, Punjab, 140413, India; c University Centre for Research & Development, Chandigarh University, Gharuan, Mohali, Punjab, 140413, India; d Department of Food Science and Technology, Guru Nanak Dev University, Amritsar 143005, Punjab, India; e Department of Physiotherapy, Guru Nanak Dev University, Amritsar 143005, Punjab, India; f Department of Food Science and Technology, Graphic Era (Deemed to be University), Dehradun 248002, Uttarakhand, India

**Keywords:** Agri-waste, Food waste, Waste-categorization, Waste management, Circular economy, Valorization

## Abstract

Agri-food waste is one of the most abundant biomass resources globally, generated through ongoing agricultural activities and food industry operations. Around 30 % of food produced for human consumption is lost during production and processing, contributing to an estimated 150 billion metric tons of waste annually—an amount projected to grow by 7.5 % each year. Conventional disposal methods such as landfilling, and incineration result in significant environmental harm and economic loss. However, agri-food waste contains valuable organic compounds that can be repurposed into value-added products, supporting the transition to a zero-waste circular economy. This review compiles recent advancements in the generation, categorization, and valorization of agri-food waste, highlighting its potential in biotechnology for producing antibiotics, fermented foods, nutraceuticals, and industrial materials. By aligning with UN Sustainable Development Goals (SDGs) 11, 12, and 13, the review underscores the need for increased research, technological innovation, and public awareness to foster sustainable waste management practices.

## Highlights

• Agri-food waste is a major global concern, with around 30 % of food lost during processing.
• Conventional disposal methods cause environmental harm and economic losses.
• Agri-food waste contains valuable organic compounds for reuse in a circular economy.
• Waste can be converted into antibiotics, fermented foods, and industrial raw materials.
• Focuses on eco-friendly strategies to reduce carbon footprints.

## Graphical abstract

**Figure:**
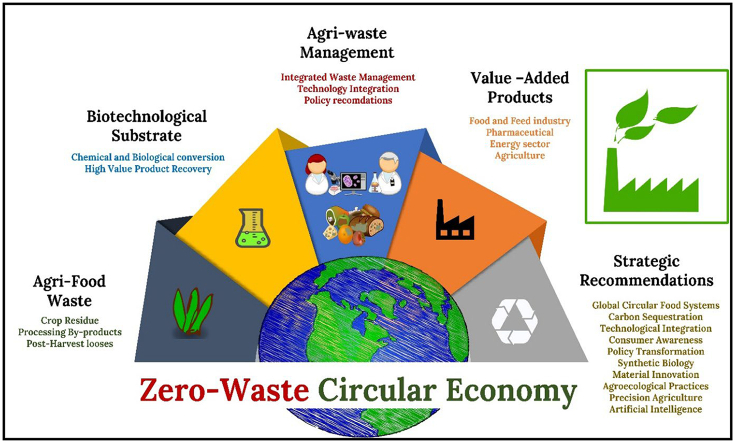

## 1 Introduction

The global population is increasing at an exponential rate and with a current gigantic figure of about 8 billion people, it is expected to reach an all-time high of nearly 9 billion by the year 2050 (Juniper, 2021). This unstoppable increase in population is putting immense pressure on various resources, including land, water, air, and food. The projections indicate that in order to feed such a massive count of people with accelerated growth rate, there will be a need of raising the global food production nearly 70 % by the year 2050 (FAO, 2025). Therefore, with a rampant increase in food production to meet the consumer needs and dietary requirements, various methods have been developed and monitored in agriculture and food processing sectors. All these production procedures generate massive amount of waste, estimated to be around 150 billion metric tons which is expected to increase with an annual growth rate of 7.5 % (Ajayi & Lateef, 2023). The steps involved in the production of food, including preparation of soil to sowing seeds, spreading and spraying fertilizers, irrigation, harvesting, processing and packaging, generate waste and runoff which enters into water, air, or soil and ultimately cause pollution. The nature of waste generated during agricultural activities could be solids, liquids, or slurries (Kaur, Singh, & Singh, 2023). Agri-food waste can constitute the components from animal wastes such as manure and animal carcasses as well as crop processing waste like fruit and vegetable peels, seeds, pomace, pruning, drops and culls, corn stalks, sugarcane bagasse. Food and Agriculture Organization (FAO) has mentioned that around 20–30 % of fruits and vegetables are discarded as waste during post-harvest handling (The State of Food and Agriculture—2019, 2025).

Most of the agri-food waste is treated with conventional methods like dumping and open-flame burning and the resultant pollution from these techniques has sanitary and environmental consequences. Combustion of crop residues as well as agri-food waste leads to the emission of toxic compounds like dioxins and heavy metals along with a massive deterioration of air quality by releasing particulate matter. In India, around 500 Mt. of agricultural residue is generated annually, of which, around 92 Mt. is burnt (Bhuvaneshwari, Hettiarachchi, & Meegoda, 2019; R. Singh et al., 2021). It also produces greenhouse gases like methane (CH_4_), carbon dioxide (CO_2_) etc., which pollute the groundwater after seeping. Landfilling causes the production of about 125 m^3^ of landfill gases, which is suggested to be the largest artificial source of CH_4_, constituting around 8 % of total CH_4_ emissions (Sridhar et al., 2021). The improper disposal may lead to air, land, and water poisoning, thereby posing serious health risks. Similarly, ineffective waste management and disposal systems endanger the inhabitants in neighboring communities (Hazell & Evans, 2011).

Agri-food waste is a rich source of organic and inorganic compounds that can be valorized to obtain highly value-added products like bio-fertilizers, biofuels, biopolymers (Oluseun Adejumo & Adebukola Adebiyi, 2021). Several studies have reported that the waste peel of fruits like pomegranate, lemon, and green walnut possess phytochemicals and have antimicrobial property which makes them a natural alternative to chemical antimicrobials (Delgado Adámez, Gamero Samino, Valdés Sánchez, & González-Gómez, 2012; Katalinić et al., 2010). Similarly, pectin isolated from fruit and vegetable waste possesses excellent film forming qualities after blending with zeolites (Phiri, Mavinkere Rangappa, Siengchin, Oladijo, & Dhakal, 2023). The waste generated from sewage sludge, kitchen, gardens, municipal solid waste (MSW), agri-industrial activity, animal farms, and so on, contain an organic biodegradable fraction and can be classified as solid organic waste (Mata-Alvarez, Macé, & Llabrés, 2000)*.* These solid organic wastes can act as a potential source for cultivating mushrooms and yielding other bio-based products, like bio-fertilizers, bio-energy, etc. Therefore, it has become necessary for researchers and policy makers to devise the appropriate strategies for reducing and reusing agri-food waste. Most of the previous review studies have primarily focused on individual techniques of agri-food waste valorization, such as composting, biofuels, renewable energy, biodegradable packaging, and phytochemical extraction. However, they often fail to present a broad overview of waste valorization in the agri-food industry. Table 1 shows the comparative analysis of different agri-food industrial waste valorization methodologies adopted by the researchers in past years and the limitation of each study in commercial applications and other sectors. This review aims to address existing gaps by providing widespread analysis of current agri-food waste disposal strategies. It explores the valorization methods adopted by major agri-food waste-producing industries, and discuss innovative techniques developed for transforming agri-food waste into valuable products. By elucidating the potential challenges and opportunities associated with valorization of agri-food waste residues, this review contributes to the ongoing discourse on the United Nations' Sustainable Development Goals (SDGs 11, 12, and 13). It promotes the principles of circular economy, explains sustainable practices, and outlines future strategies for effective waste management in agriculture and allied sectors.

**Table 1:** Comparative analysis of the studies adopting agri-food industrial waste valorization, highlighting the findings, methodologies, and limitations of each study.

**Study**	**Objective**	**Methodology**	**Key Findings**	**Limitations**
Asagbra et al., 2005	Production of tetracycline using agricultural wastes as substrate.	Solid-state fermentation of peanut shells, corncob, corn pomace and cassava peels using different strains of Streptomyces.	- *Streptomyces* sp. OXCI produced highest amount of tetracycline with peanut shells (13.18 mg/g) - Peanut shells were the most effective substrate followed by corncob, cassava peels, and corn pomace i.e., 2.64 mg/g, 2.16 mg/g, and 1.99 mg/g, respectively.	- Optimization of substrates and strains is required for higher efficiency. - Further study on mixed agri-food waste as substrate need be checked for commercial application. - Trained manpower and high installation cost.
Vastrad & Neelagund, 2011	Utilization of pineapple peel as a novel substrate for the production and optimization of tetracycline.	Solid substrate fermentation of pineapple peels with various strains of *Streptomyces.*	- Pineapple peel can be used for the production of tetracycline as solid substrate. Addition of inorganic salts, organic and inorganic nitrogen source improve the yield of tetracycline.	- The cost effectiveness of producing tetracycline using pineapple peel as solid substrate need to be identified for commercial application.
Antónia Nunes et al., 2019	Valorization of olive pomace by a green integrated approach.	An environment friendly integrated approach was applied to extract and concentrate the phytotoxic compounds of olive pomace using three types of polymeric composite membranes.	- BW 30 membrane was found most effective in concentrating the extract with no phenolic compounds in the permeates	- Study is limited to extraction and concentration of toxic phenolic compounds. - Further application of toxic phenolic compounds need to be identified. - There is no provision for utilization of solid waste after extraction.
Muñoz, 2019	Anaerobic digestion of food waste at psychrophilic range to reduce environmental impact.	Batch and Semi-continuous anaerobic digestion were performed with different food waste to inoculum ratios.	- Increase in food waste and inoculum ratio significantly reduced the specific methane yield (SMY) (up to 65 %) while soluble chemical oxygen demand (SCOD) remains quite constant. - In semi continuous operation SMY and SCOD reduced up to 70 and 73 %, respectively with increasing organic load rate.	- Experiment was conducted at Lab scale under air-conditioned room. - Study conducted with only a 5 L glass reactor digester. - Study is limited to a batch or semi continuous type of digestion. - Disposal of remaining solid waste has not been discussed.
Simonato et al., 2019	Fortification of pasta with olive pomace.	Formulation of pasta by replacing 5 or 10 g/100 g semolina with olive pomace.	- Fortification increased the total phenolic content and antioxidant properties of pasta. - Decreased cooking time, and increased swelling index. - Increased the firmness of pasta. - Increased slowly digestible and resistant starch without affecting glycemic index.	- Very limited amount of olive pomace fortification is possible due to change is sensory properties of pasta. - Consumer study need to done to assess the commercial application.
Bender et al., 2017	Effect of grape pomace skins in technological properties of muffins.	Formulation of muffins using grape pomace skin as a partial replacement of wheat flour.	- Soluble dietary fiber content of muffins was increased significantly. - Instrumental parameters (color, and texture) of muffins affected significantly. - Consumers didn't perceived any substitution in the formulated muffins.	- Changes in phytochemical composition was not identified. - Consumer study was limited to lab scale. - Change in dietary fiber of muffins depends on skin of grape variety used.
Kumar et al., 2025	Bio-ethanol production form waste potato using ultrasonic-assisted acid hydrolysis	- Optimization of fermentation process using *Saccharomyces cerevisiae*, and ultrasonic assisted acid/enzymatic hydrolysis.	- The ultrasonic-assisted acid/enzymatic hydrolysis increased TRS production by 10.6 % i.e., 116 g/L in case of conventional hydrolysis. - Ultrasonic-assisted staged acid/enzymatic hydrolysis cum saccharification increased the TRS by 29.17 %. - Maximum 61.8 g/L ethanol content was achieved.	- The availability of raw material is very limited and other agri-food waste also need to be tested for commercial application.
Caballero et al., 2020	Enhancing polyphenolic compounds extraction from olive waste using Supercritical Fluid Extraction (SFE)	- SFE of olive residues, i.e., pruning biomass, leaves and exhaust pomace at different pressure level.	- SFE at 300 bar shown highest extraction efficiency of antioxidants and hydroxytyrosol as prominent compound from olive leaves, pomace and pruning biomass.	- Application of solid mass after extraction of phenolic compounds need to be explored. - Commercial application of phenolic extract need to be identified.
Kumanda et al., 2019	Valorization of red grape pomace waste through formulation of functional feed for broiler diets	- Formulation of experimental diets for growing and finishing chickens with polyethylene glycol (PEG) and enzyme treated red grape pomace.	- PEG significantly improved the nutritional quality of feed and reduced anti-nutritional effects of condensed tannins. - Cellulolytic enzyme treatment was ineffective in improving nutritional quality of formulated feed.	- The fiber and condensed tannins found in red grape pomace reduce the digestibility of grape pomace-based feed in chickens. - Poor bio-availability of bioactive compounds.
Cruz et al., 2020	Removal of arsenic and lead form water using agrowaste derived biochars impregnated with ZnO	- Biochars was derived by carbonation of corn cob and coffee husk under mind conditions along with impregnation with ZnO using precipitation method.	- ZnO impregnation improved the adsorption capacity of derived biochars. - Corncob derived ZnO impregnated biochar shown maximum adsorption of As and Pb in polluted water.	- Impregnation of ZnO in biochars need to be optimized. - Impregnation of biochars with other coating material need to be checked for better adsorption.
Vardon et al., 2013	Complete valorization of spent coffee grounds (SCGs) in the form of biodiesel, bio-oil and biochar	- Methodology involves extraction of lipids, and defatted grounds to produce biodiesel, bio-oil and biochar from SCGs through transesterification and slow pyrolysis.	Coffee biodiesel blend with ultralow sulfur petroleum diesel (ULSD) significantly improved the fuel properties as per the standards prescribed by ASTM D975 and D7467. - Slow pyrolysis of defatted SCGs resulted 13.7 % bio-oil yield. - The biochar solids yield was comparable for spent and defatted grounds i.e., 27–28 %. - Coffee-derived biochar can be used in combination of fertilizer for soil amendment to increase biomass yield of crops.	- Several properties of produced coffee biodiesel don't meet the standard prescribed by ASTM D6751 or EN 14214. - Poor oxidative stability of biodiesel - Coffee biochar did not increase the biomass productivity if used without combining with fertilizers. - Further work needed to determine the mechanism behind linkage between physico-chemical properties of coffee biochar, fertilizer efficiency, quality of soil and crop yield.
Dutournié et al., 2019	Valorization of olive mill wastewater as a green fuel, agricultural water source and bio-fertilizer	- Impregnation of wood sawdust with olive mill wastewater (OMWW). - Drying and pyrolysis of impregnated samples.	- About 95 % water can be recovered from OMWW - Low electrical conductivity and high pH of condensed OOWW. - Low-cost additional treatment of water needed for reuse in agriculture.	- The recovered olive oil mill water has acidic pH value and slightly high chemical oxygen demand. - Additional treatment to recovered water is required before reuse in agriculture.
Selvam et al., 2014	Production of cellulase from coffee pulp and pineapple waste by alkali treatment using *Acinetobacter* sp.	- Optimization of cellulase production in SSF using newly isolated species of *Acinetobacter* TSK-MASC	- Highest production of cellulase i.e., 888 U/mL was achieved after 60 h of incubation with 3.0 g/L of coffee pulp waste and pineapple waste at pH 7.0.	- Further purification of enzyme needed. - Cellulase production waste not compared with other studies. - Economical aspects need to be checked for commercial application. - Provision of disposal of residual waste after SSF also need to be developed.

## 2 Categorization and utilization of Agri-food waste

**Fig. 1:**
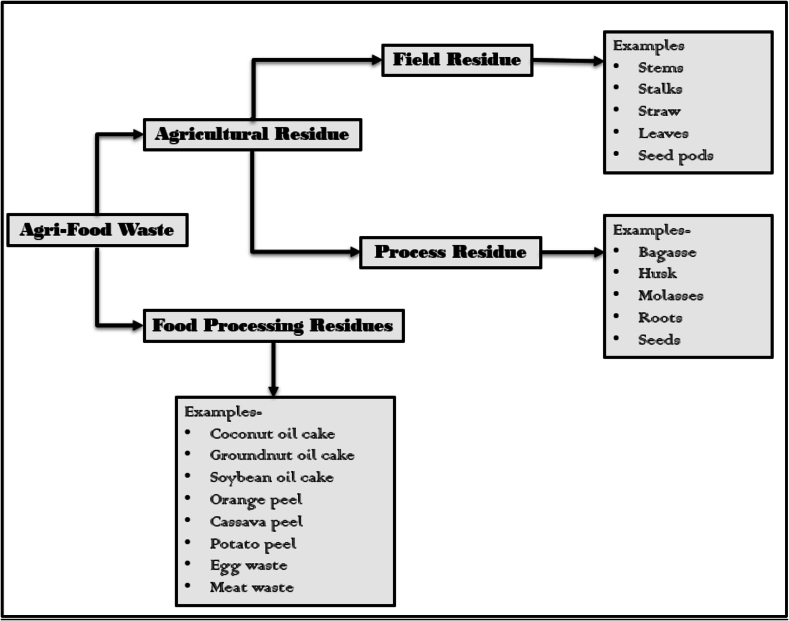
Classification of Agri-Food Waste.

Agri-food waste is generated during food production, processing, packaging, storage, transport, spoilage, and discarding. One way of classifying agri-food waste is based on the source of generation. Fig. 1 depicts two types of agri-food waste, including residues generated by agricultural activity and food processing. Field residues and process residues are the two further fractions of agriculture residues. Field residues can be defined as the residues that remain in the field after harvesting of crops, including leaves, stalks, straw, seed pods, and stems. In contrast, process residues may include the waste generated during crop processing to obtain any valuable product. On an average, the amount of agri-food waste produced annually from agriculture and food processing operations has reached 1.3 GT globally (T. T. T. Nguyen, Malek, Umberger, & O'Connor, 2023; Onu & Mbohwa, 2021). The volume of agri-food waste generated can vary depending upon the type of crop and the nature of its processing. For instance, the waste can be as low as 12 % in case of coffee and can reach up to 85 % of the total produce in the case of corn processing (Cruz et al., 2020; Porichha, Hu, Rao, & Xu, 2021).

### 2.1 Agricultural residues

#### 2.1.1 Field residues

Cereal straws are the major type of field residues, including sugar crops (sugar beet, sugarcane, sorghum fiber, and sweet sorghum), starch crops (wheat, barley, and corn), and oil crops (Chakrabarti, Khan, Kishore, Roy, & Scott, 2019). After harvesting grains from cereal plant, the left-over dry stalk is known as cereal straw. A limited volume of this straw is utilized for feeding and bedding animals, compost for mushroom cultivation, or as a garden mulch after harvesting, while the remainder is either burned or integrated into the soil. Burning at the field has been a standard straw disposal method for years. This conventional disposal technique is hazardous to the environment and poses a significant risk factor for respiratory diseases (Chakrabarti et al., 2019). However, numerous countries have executed bans on this practice due to the pollution it generates, a lack of implementation of such government policies is witnessed at various regions in India (Cruz et al., 2020). Therefore, compaction technologies have been developed to manufacture high-energy density straw pellets and wafers (Yunaidi, & F., & Harnowo, S., 2020). Researchers reported that the residue of wheat straw furnishes 11.8–71.2 l/Kg of bio-ethanol, 298 l/Kg of bio-methane, and 1–68 ml of bio‑hydrogen per gram, while barley straw can yield 11.9–46 l/Kg of bio-ethanol on bioprocessing (Onu & Mbohwa, 2021).

Corn stover are the leftover non-grain above-ground parts of the plant, including stalk, tassel, leaves, husk, cob, and silk. A corn stover does not include the crown and surface root parts of the plant. About 5 % of the stover is used for bedding and feeding animals, while the rest is plowed into the soil. Corn stover has the potential to be burned directly, converted into biofuels, and used as fiber in the pulp and paper, particle board, and/or oriented strand board sectors (Savoie & Descôteaux, 2004). Similarly, corn stalk has been reported to yield bio‑hydrogen upon fermentation (Onu & Mbohwa, 2021). Some studies have reported to use the corn-based agri-waste to produce poly-lactic acid, an effective biopolymer with a high ultraviolet barrier (Phiri et al., 2023). Rice straw has shown to have a potential of producing 12–29.1 l/Kg of bio-ethanol, 302 l/Kg of bio-methane and 24.8 ml/g of bio‑hydrogen (Cruz et al., 2020).

#### 2.1.2 Process residue

Under the category of process residues, husk from rice crops is the most prevalent type due to its vast global production. Around one ton of husk is produced for every four tons of rice grains. In rice processing factories, husk is removed from the rice grains, and the biomass is collected. Unlike other forms of biomass, the rice husk is more homogeneous and possesses better flow characteristics. As a result, rice husk can be used in biofuel technologies like gasification, where homogeneous fuel quality is desired for better performance. However, husks contain high silica concentrations, which can produce ash and slag in the boiler during combustion (Yunaidi, & F., & Harnowo, S., 2020). Rice bran, which is generally removed during second milling process of rice, has been reported to produce biodiesel by following a two-step in-situ transesterification process (Shiu, Gunawan, Hsieh, Kasim, & Ju, 2010). Residues from the processing of corn (i.e. corncob hydrolysate and corn steep liquor) have been shown to produce butanol by fermentation using *Clostridium beijerinckii* (Zhang & Jia, 2018).

Sugarcane is one of the most efficient photosynthetic plants with the highest bioconversion efficiencies to fix solar energy. The sugarcane harvesting leaves behind the energy rich fibrous bagasse after the extraction of juice for further conversion to sugar (Kabeyi, 2020). In most sugar mills, sugarcane bagasse generates the heat required to boil sugarcane juice and to concentrate syrup. Moreover, large sugar mills have practiced burning waste bagasse for energy requirements as a method of disposal when in excess at the plant. The heating value of sugarcane bagasse (on dry basis) is approximately 19.2 MJ/kg (Yunaidi et al., 2020). With the action of *Clostridium acetobutylicum*, sugarcane bagasse has been shown to produce bio-butanol (Abd-Elsalam, Periakaruppan, & Rajeshkumar, 2021). In a similar manner, several pre-treatment methods like milling, acid- and enzyme-hydrolysis and combined treatments have been used to enhance the breakdown of process residues for conversion into inexpensive substrates and value-added products (Lopes & Ligabue-Braun, 2021).

### 2.2 Food processing residues

Several studies have reported that the pattern of food wastage varies, depending upon the monetary status of the country (Rahman et al., 2024)**.** In underdeveloped countries, primary food wastage occurs due to a lack of infrastructure, insufficient provisions for waste collection and facilities to preserve the entire agricultural produce till it is finally retailed (Das et al., 2019). In contrast, utilization and discarding of food at domestic and industrial levels are the primary reasons for food wastage in developed countries (Rahman et al., 2024)**.** Apart from the food industry, domestic household activities, restaurants, hotels, and retail shops are prominent contributors to food residue generation (Dhir, Talwar, Kaur, & Malibari, 2020). This trend of food wastage is estimated to increase in the coming years, with the predicted 60 % upsurge in food demand by 2050 (*Food Wastage Footprint—Impacts on Natural Resources – Summary report, 2025*). Other than causing economic and social losses, food wastage is also a substantial reason for environmental degradation.

Food processing residues are a significant component of agri-food industrial waste, generated during the processing and packaging of plant and animal products. These residues include a wide variety of organic materials such as fruit and vegetable peelings, pulp, and pomace, as well as waste from slaughter houses and dairy operations (Barati, 2023). This category of residues is a rich source of carbohydrates, proteins, and bioactive components like, phenolic acids, flavonoids, and carotenoids. These also contain valuable nutrients like nitrogen, potassium, and phosphorus, which can enhance soil fertility (Barati, 2023). The cakes/meals left after the extraction of oil from diverse food sources like, oilseed, soyabean, coconut, groundnut etc. are the enriched sources of protein. These residues have been utilized to extract protein isolates, bioactive peptides and can be used as substrate for value-added product formulations (Ghosh, Khuntia, & Mitra, 2018).

## 3 Current approaches to Agri-food waste management

Waste management represents one of the significant environmental challenges encountered worldwide. An increase in waste generation by human emissions and changes in consumption patterns have led to higher solid waste generation rates. Managing waste involves dealing with solid, liquid, gaseous, or radioactive substances, requiring specific methods and expertise.

A typical waste management system includes the collection, transportation, pre-treatment, processing, and final disposal of waste. The primary goal of waste management is to create sanitary living conditions by minimizing the garbage generation while encouraging the recycling of waste materials. Waste can be recycled in a variety of ways, such as, extracting valuable resources, reprocessing, or treating thermo-chemically to produce electricity. Composting is yet another commonly used method of agri-food waste management. A recent study has claimed an energy recovery rate of up to 2,04,907 kJ/h via composting on the commercial scale (Sridhar et al., 2021). Pyrolysis is an emerging technology that can obtain value-added products like biochar and biodiesel from agri-waste. Moreover, agri-food waste can also be bio-processed to obtain several bioactive compounds like phenols, enzymes, pigments (Sridhar et al., 2021).

**Fig. 2:**
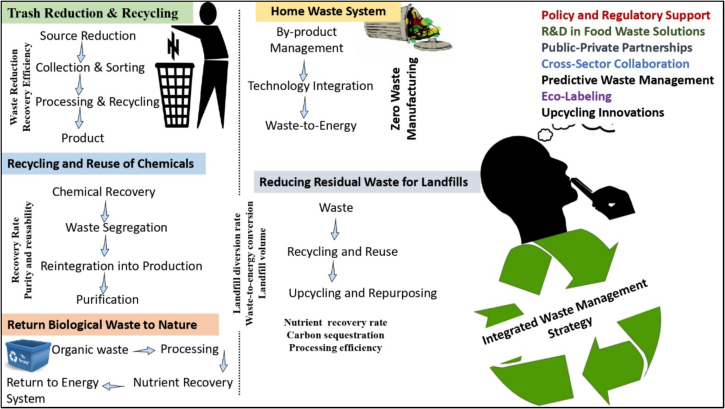
Integrated Agri-Food Waste Management Strategies.

The waste management situation in developing countries differs from that of developed nations. Transferring proven waste management technologies from one country to another may be theoretically feasible, economical, and sustainable, but its effectiveness in developing nations can vary. Therefore, it is essential to understand local factors such as waste characteristics and seasonal climate variations, social dynamics, cultural attitudes toward waste, and political structure. Additionally, awareness of the resource limitations that often exist in these regions is crucial for successful implementation of the waste management strategies. In India, the vast majority of food waste is processed conventionally, through the methods like burning, disposing off in landfills, composting, and anaerobic digestion, which yields products like water gas, biodiesel, bioethanol, syngas, pectin, cellulose, fiber and other microbial metabolic products that are used as industrial raw materials (Fig. 2).

### 3.1 Landfilling

One of the most popular techniques for removing food waste is landfilling. It entails placing food waste in a pit and covering it with dirt, sand, or debris. Despite being inexpensive and simple to transport, this technique poses substantial risk to the air, water, land, and environment. The biodegradable waste in landfills decomposes with time and generates CH_4_ and CO_2_, which are greenhouse gases, contributing to the global warming (Maraveas, 2020). The approach of recycling of municipal solid waste (MSW) and waste-to-energy strategies are the promising approaches to mitigate CH_4_ emission from MSW (Matthews & Themelis, 2007). However, the primary issue with such methods of disposing agri-food waste is their large carbon footprint, poor conversion efficiency, and noteworthy loss of valuable substances which may have adverse medical and financial consequences.

### 3.2 Incineration

Incineration is considered as a simple method of garbage treatment which involves combustion of trash in the presence of oxygen while reducing or maintaining the emission levels below current emission standards and producing energy (Buekens, 2013). Along with that, the suitable levels of temperature, turbulence and residue time plays role in achieving waste volume reduction using incineration (Buekens, 2013). The heat produced in this manner can be used thermally as a source in turbines, heat engines, boilers, power plants, etc. Moreover, the valorization of bottom ash left after the incineration of municipal solid waste is next possible method of treating harmful fly ash, a secondary waste after incineration (Bogacka, Poranek, Łaźniewska-Piekarczyk, & Pikoń, 2020). Further, the alkaline pre-treatment of this secondary waste helps in improving the waste quality, hence, justifying the norms of circular economy and sustainable development (Czop, Poranek, Czajkowski, & Wagstyl, 2019).

### 3.3 Composting

Microbiological regeneration through composting provides a sustainable alternative for agri-food waste management by converting the organic waste into nutrient-dense biofertilizers. This solution helps in enhancing the soil health, and reducing the environmental pollution. A diverse range of microorganisms are introduced during the intricate biochemical process of composting, which helps to treat agri-food waste. These microorganisms generate valuable products like organic acids and bioethanol from raw biomolecules found in biodegradable waste. Livestock manure and agricultural waste like, wheat straw, rice husks are decomposed by the action of microorganisms viz., bacteria (e.g. *Azospirillum*, *Pseudomonas*) and fungi. The breakdown of complex organic matter into humus by these microbes provides a source of nutrients to soil (Ayilara, Olanrewaju, Babalola, & Odeyemi, 2020; Imran et al., 2022). Compost is generally augmented with plant growth-promoting rhizobacteria (PGPR) to produce bioorganic fertilizers. Such microbes improve nutrient availability, produce phytohormones, and enhance root proliferation, leading to a better water and nutrient uptake in plants (Imran et al., 2022). It has been observed that the compost enriched with microbes helps in improving soil fertility, moisture retention along with reduction in soil erosion (Zainudin, Mohd Yusoff, Ramli, & Mohd Yasin, 2024). The compost also helps in retaining the organic carbon content of soil, thus reducing the emission of greenhouse gases (Jeong, Cho, Lee, Kim, & Kim, 2019; M. J. Nazir et al., 2024).

### 3.4 Anaerobic digestion

Anaerobic digestion (AD) is a most promising biochemical process of converting agri-food waste into renewable energy and nutrient-dense fertilizers by way of microbial activity in oxygen-free environment. AD has been considered as a best approach of addressing the challenges of waste management while reinforcing circular economy norms. AD provides an ecological atmosphere with a consecutive breaking down of complex organic polymers of food waste into soluble organics, which further gets converted into biogas by microbial action (Hatti-Kaul & Mattiasson, 2016; Singh, Solanki, Ghosh, & Pal, 2020). CH_4_, CO_2_, and Hydrogen are produced along with the degradation of organic polymers under the action of anaerobic microbial metabolic activities (i.e. hydrolysis, acidogenesis, acetogenesis, and methanogenesis) on processed food waste (Czatzkowska, Harnisz, Korzeniewska, & Koniuszewska, 2020). The process of breaking down complex organic polymers of food waste into simpler sugars and fatty acids is known as hydrolysis. This is followed by acidogenesis, which is the process of turning complex polysaccharides, proteins, and fats into intermediate fermented products; acetogenesis, which is the process of turning high-order fatty acids into acetate compounds and Hydrogen; and methanogenesis, which is the process of turning acetates into CH_4_ (Muñoz, 2019).

## 4 Waste generation and valorization approaches in key Agri-food processing industries

Agri-food waste generation is a major global challenge where several industries contribute in a way or other, leading to the contamination of natural resources. Around 7.5 billion tons of food in different forms is generated annually to feed the massive global population (Global food losses and food waste, 2025). Still, more than one-tenth of the human population remains hunger-stricken. According to the latest UNEP (United Nations Environment Programme) Food Waste Index Report 2024, one-fifth of food produced for the intention of human consumption gets wasted annually, leading to a global economic loss of 1 trillion USD (Environment, 2024).

Waste generated from industries often contains valuable nutrients and bioactive compounds that can be utilized in the production of functional compounds or biofuels. An enormous body of research targets sustainable methods of handling food processing residues. The objectives of SDGs and circular economy necessitate the reduction of per capita food waste generation to half by 2030. Advanced marketing techniques like valuation or discount offers on soon-to-be expired food products can boost sales and prevent food losses from retail outlets. Green practices involving voluntary food donations before expiration can also reduce food waste generation which could be a great contribution toward United Nations' SDGs 11, 12 and 13 (Dou et al., 2016; Hayashi, Homma, & Akimoto, 2022; Islam, Nazir, & Rahman, 2023). The major industries contributing to agri-food waste generation, along with their potentially important residues as a valuable substrate for the valorization are addressed below.

### 4.1 Wine processing industry

The beverage industry is considered to generate the highest proportion of agri-food waste, accounting for nearly 26 % of total food waste globally (Matei et al., 2021). This includes by-products such as spent grains, fruit pomace, and liquid effluents. Wines are among the widely accessible alcoholic beverages around the world, and the primary waste product of the wine industry contains grape skin, pulp, and seeds (Xavier Machado, Portugal, Padilha, Ferreira Padilha, & Dos Santos Lima, 2021). Grapes are one of the most extensively cultivated fruit crops, with an annual production of around 77.8 million tons (Ilyas et al., 2021). In every harvesting season, approximately 44 million tons of grapes are used to produce juice and wine. The amount of grape pomace generated from wine-making depends upon the grape species, processing/pressing conditions, and type of equipment used. Surprisingly, the bisphenols available in grape fruits are also found in wine and juice processing residues. Among others, anthocyanins, procyanidins, flavonoids, and stilbenes are the most abundant polyphenols in grape pomaces and have vital biological functions (Abbasi-Parizad et al., 2021; Xavier Machado et al., 2021). The application and significance of different modern extraction methods has been studied for the extraction of bioactive compounds from grape by-products. The pressurized liquid extraction method was found effective for recovery of maximum phenols from grape by-products (Perra et al., 2023). The application of pulsed electric field in isolation of bioactive glycosylated and lipidic compounds with high antioxidant activity (68 %) was reported earlier (Salgado-Ramos et al., 2023). Similarly, enzyme-assisted extraction technique was found efficient in the extraction of phenolic compounds from grape pomace, followed by the extraction using acetone (Ferri et al., 2023). In another study, grape pomace was observed as a suitable substrate for the production of microorganisms using enzymatic treatment, which ultimately help in breaking down of high-molecular weight compounds of pomace (Ageeva, Tikhonova, & Globa, 2022). The valorization and treatment of the by-products of wine making (e.g., grape marc) could be done by anaerobic digestion, composting, and vermin composting at a pilot and industrial scale (Gómez-Brandón, Lores, Insam, & Domínguez, 2019). In a recent study on the valorization of grape pomace from the wine industry, anaerobic digestion was observed to be a good choice due to low greenhouse gas emissions and tannin fabrication and also considered as the highly balanced approach presenting optimal economic and environmental benefits (Taifouris, El-Halwagi, & Martin, 2023).

The value-added utilization of grape pomace has been widely documented. The use of grape pomace as poultry feed was reported to increase meat quality (Erinle, Oladokun, MacIsaac, Rathgeber, & Adewole, 2022), meat yield (Kumanda, Mlambo, & Mnisi, 2019), along with feed cost reductions (Navidshad & Royan, 2025). The addition of grape pomace with manure showed a major impact in preserving soil nutrients due to high polyphenol content of pomace (Korz et al., 2023). Further, the incorporation of grape pomace in food formulations has shown the health promoting effects. The fortification of foods with grape pomace resulted in an increase in antioxidant activity (Maestre, Micol, Funes, & Medina, 2010), dietary fiber content (Choi et al., 2010) and an improvement in fatty acid profile (Bender et al., 2017). Also, the antimicrobial activity of grape pomace against food spoilage microbes has been reported (Lorenzo, Sineiro, Amado, & Franco, 2014).

### 4.2 Milk processing industry

Dairy waste contributes around 21 % of global food waste. Milk by-products produced during the processing include cheese whey, dairy scum, whey permeates, curd clumps, and spilled or expired milk. Due to the presence of milk remnants, these by-products are considered dairy pollutants (Lorenzo et al., 2014). The high organic load of waste water from dairy industry due to the presence of proteins, fats, and carbohydrates in milk has been reported, and this may vary depending on the product being processed (Ahmad et al., 2019). The milk processing industry generates between 115 and 180 million tons of whey per year, of which roughly 47 % *w*/w is discarded in the environment, posing major pollution due to its high biological oxygen demand (BOD) and chemical oxygen demand (COD) (Wen-Qiong, Yun-Chao, Xiao-Feng, Rui-Xia, & Mao-Lin, 2019). Cheese and casein manufacturing generates a large quantity of whey which is a major by-product of dairy industry, containing vitamins, lipids, and carbohydrates (Mehra et al., 2021). Being rich in organic matter, whey poses an additional burden on sludge treatment systems due to high BOD and COD, and if disposed of untreated, it can pollute the environment. Researchers analyzed the physicochemical properties of cheese whey and observed that it contains approximately 93 % water, 0.3 % fat, 0.8 % protein (representing 20 % of total milk protein), 4.8 % carbohydrate (lactose), and 0.5 % (*w*/w) ash, all of which can be further utilized for making value-added products (A. Nazir et al., 2019).

Therefore, recent studies are focused on utilizing solid waste (sludge) and waste water (effluent) from the dairy industry for producing single-cell protein, biofertilizers, energy, and bio-products (Adesra, Srivastava, & Varjani, 2021; Pugazhendhi et al., 2019; Rene, Ge, Kumar, Singh, & Varjani, 2020). The production of biofuels (Hydrogen and CH_4_) from dairy waste is an innovative approach of creating an alternative clean energy source for sustainable development (Kazmi et al., 2024). AD is considered as the most promising method of dairy waste management, where the carbohydrates, proteins, and fats present in dairy waste work as essential nutrients for anaerobic bacteria, leading to biogas production (Prakash, Sharma, Patel, Kim, & Kalia, 2018; Saadia, Houcine, Abdelhak, & Ahmed, 2025).

### 4.3 Olive processing industry

In Mediterranean regions**,** olives are believed to be among the first and most crucial farming products. Olive pomace utilization has expanded over the past few decades, with a periodic global production exceeding 3Mt (Antónia Nunes et al., 2019). The rising utilization of olive pomace and olive pomace oil is due to the scientific validation of its health promoting properties. As Mediterranean countries account for nearly all of the world's olive production, the industry is economically profitable and hold substantial environmental, and social significance in the region. Large quantities of wastewater and sediments are generated during the processing of olives to obtain olive pomace oil. The choice of extraction method depends upon the olive oil waste composition, kind and age of olives, meteorological and agricultural conditions of the region (Di Lecce et al., 2014; Sedej et al., 2016). The conventional method like maceration and green technology extraction methods like, infrared, microwave- and ultrasound-assisted extraction have been used for olive oil extraction, where these methods give better extraction yield with lesser solvent utilization and minimum component degradation as compared to that of conventional methods (Rosa et al., 2021). Also, pressurized liquid extraction (Rosa et al., 2019) and super-critical fluid extraction (Caballero, Romero-García, Castro, & Cardona, 2020) methods were operated for the extraction of total phenolic compounds from olive oil production by-products with variable extraction yield.

The olive oil business is progressively moving away from the old three-phase manufacturing system to favor a two-phase approach, which produces less effluent during processing and is more environment friendly. Olive pomace produced by two-phase milling system is a semi-solid slurry which contains pieces of olive skin, pulp, stone (solid phase), water, and oil (Antónia Nunes et al., 2019). This slurry has a more considerable COD than a three-phase system, making it more polluting if discarded (El Yamani, Sakar, Boussakouran, Ghabbour, & Rharrabti, 2020). The conventional three-phase olive mill production system is a water-intensive process (Di Lecce et al., 2014), generating substantial amounts of dark liquid effluents known as olive mill waste waters (OMWWs). This waste water is characterized by high electric conductivity (9.7 13.8 ms/cm), a pH of 4.8–5.6, and an average composition of 83–92 % water, 4–16 % organic compounds, and 1–2 % (*w*/w) inorganic salts (Dutournié, Jeguirim, Khiari, Goddard, & Jellali, 2019; El Yamani et al., 2020). Organic acids like citric-, malonic-, lactic-, tartaric-, succinic-, and fumaric-acid contribute to the low pH of OMWWs (Papaioannou et al., 2022). Water used for the washing of olives (i.e., approximately 5 % w/w of the processed olives), olive pulp water (i.e., 40–50 % w/w of the initial weight of olives), water added during olive paste centrifugation step, and water from the washing of equipment (i.e., 5–10 % w/w of the load of the processed olives) have been considered as the potential sources of OMWWs. The COD and BOD of OMWWs ranges from 40 to 220 g/L and 35–110 g/L, respectively (Cassano et al., 2013). Furthermore, one cubic meter of untreated OMWWs is estimated to have a pollution level comparable to 100–200 m^3^ of household sewage (Di Lecce et al., 2014). During the processing of olives, only a tiny fraction of bio-phenol (2 % w/w total phenolic content (TPC) penetrates the oil phase (De Marco, Savarese, Paduano, & Sacchi, 2007), due to their physicochemical properties. Whereas the majority of these bio-phenols leach in waste waters (53 % w/w TPC) and pomace (45 % w/w TPC) in three-phase olive mills (De Marco et al., 2007) or in a semi-solid slurry when processed by two-phase olive mill (Antónia Nunes et al., 2019). TPC in three-phase systems typically ranges from 1.6 to 10.7 g GAE/L, based on the amount of water injected throughout the process. More than 50 bio-phenols have been identified in OMWWs, with hydroxyl tyrosol and tyrosol being the most prevalent constituents (Tundis et al., 2021). It is important to note that the composition of bio-phenols in untreated OMWWs is primarily influenced by their residence period. High molecular weight (MW) bio-phenols (e.g. hydroxyl tyrosol and tyrosol), can be broken down by enzymes or bacteria, resulting into low MW bio-phenols. However, the oxidation of phenols can cause the opposite reaction, resulting in larger, more significant MW molecules. This implies that an appropriate treatment process for OMWWs must be developed to recover the specific bio-phenol.

The residual olive oil present in olive pomace is of high economic value and can be recovered by using solvent extraction methods (El-Emam, 2023). A similar study was conducted by Mehdi, Naqvi, Khan, and Mirani (2023) for optimising the oil extraction process using response surface methodology (RSM). The researchers considered extraction time, solvent-solid ratio, and particle size as major factors determining the yield of olive oil from olive pomace. RSM assisted in identifying extraction time of 2 h, 30:1 of solvent-solid ratio and 0.1 mm of particle size which yielded a maximum of 12 % oil (Mehdi et al., 2023).

In addition to oil, the utilization of olive pomace for the sustainable development of various products in cosmetics, pharmaceuticals and food sectors has been observed due to the presence of essential bioactive components (Antónia Nunes et al., 2018; Miralles, Chisvert, & Salvador, 2015). The olive fruit is considered as an enriched source of several nutrients viz., minerals, lipids, dietary fiber and oligosaccharides, so is the olive pomace, along with high phenolics and vitamins (fat-soluble) (Araújo, Pimentel, Alves, & Oliveira, 2015). High antioxidant property of olive pomace due to the presence of phenolic components suggests its utilization for value-added product formulations (Araújo et al., 2015). Food fortification with olive pomace showed an increase in fiber, carotenoids and phenolic content etc. in the final product (e.g. pasta) (Durante et al., 2019; Simonato, Trevisan, Tolve, Favati, & Pasini, 2019). Further, the polar lipids present in olive pomace exhibit the anti-thrombotic (Tsoupras, Fragopoulou, Iatrou, & Demopoulos, 2011) as well as anti-atherosclerotic activities (Ntzouvani et al., 2021) by preventing platelet aggregation. The cosmetic applications of olive pomace have been widely explored due to the impact of macronutrients (oligosaccharides and pectins) of pomace in improving the physical characteristics, viscosity, oxidative stability, and structure of skincare products (Rodrigues, Pimentel, & Oliveira, 2015). Additionally, the sugars viz., cellulose, hemicellulose, mannitol present in olive pomace plays a vital role in improving the oil holding capacity of cosmetics (Lo Giudice et al., 2021). The utilization and applications of by-products from olive oil processing industry have been studied in biomedical field, and nutraceutical product formulations with an innovative strategy of achieving consumer expectations along with human wellbeing and environmental safety (Otero et al., 2021).

### 4.4 Coffee processing industry

Coffee is one of the most popular beverages consumed worldwide and is the second most valuable commodity, next to petroleum (Murthy & Madhava Naidu, 2012). Coffee beans are the seeds located inside coffee cherries (usually two per cherry) and surrounded by different layers of endosperm, endocarp, mesocarp, and epicarp, from the bean to the fruit's outer skin. The beans are directly connected to the first two layers viz., silver skin and parchment. Parchment is a fibrous membrane composed of cellulose (40–49 %), hemicellulose (25–32 %), lignin (33–35 %), and ash (0.5–1 %) (Acosta-Fernández, Poerio, Nabarlatz, Giorno, & Mazzei, 2020). The composition of coffee cherries illustrates that coffee parchment, silver skin, and spent coffee grounds (SCGs) account for 35–61 g/kg, 42 g/kg, and 650 g/kg of the fresh coffee cherry, respectively (Hejna, 2021). The worldwide production of coffee waste from the coffee industry is around 10 million tons annually (Jasim, 2021). Husk, pulp, and mucilage are some other forms of coffee waste than silver skins and SCGs, produced at various coffee processing stages, including harvesting, processing, roasting, and brewing (Lestari, Hasballah, Listiawan, & Sofia, 2022). The average coffee waste includes oils (7.9–26.4 %), crude fibers (19.7–22.1 %), and various other phytochemicals, including alkaloids, polyphenols, and their esters, etc. Some of these phytochemical residues possess bioactivity and can be recovered by using the combination of right technologies (Jasim, 2021). The chemical composition of coffee waste, like other natural commodities, is heavily influenced by the origin and variety of beans, roasting conditions, extraction method, and type of by-products (Arya, Venkatram, More, & Vijayan, 2022).

A lab-scale study on the application of coffee industry by-products has demonstrated that coffee bean waste can be used as a bio-adsorbent to remove heavy metals and other pollutants (Skorupa, Worwąg, & Kowalczyk, 2023). Moreover, the use of SCGs as an affordable bio-sorbents for removing dyes, heavy metals, and pollutants from liquid waste has also been analyzed (Anastopoulos, Karamesouti, Mitropoulos, & Kyzas, 2017). Coffee silver skin is a rich source of antioxidants, polyphenols, dietary fiber, and polysaccharides (cellulose and hemicellulose) (Selvam et al., 2014). In some regions of the world, the coffee silver skin has been utilized as an additive in soil fertilizer or fuel (Saenger, Hartge, Werther, Ogada, & Siagi, 2001). Coffee husk was found to have the highest xylanase (Taifouris et al., 2023) and cellulase (Selvam et al., 2014) activity in solid-state fermentation (SSF) when processed using *Penicillium* sp. and *Acinetobacter* sp., respectively. Coffee husks and pulp are considered as a rich source of minerals, especially potassium, due to which this residual waste can be used as organic fertilizer, primarily in potassium-deficient soils (Campos, Pinto, Melo, Da Rocha, & Coimbra, 2021). The potential of SCGs in bioethanol production has been widely studied, and modifications in techniques for oil extraction from SCGs have been proposed for economically feasible production of bio-ethanol (Tuntiwiwattanapun, Monono, Wiesenborn, & Tongcumpou, 2017). Other value-added products like bio-sugars (in the form of D-mannose), due to the presence of high mannose content in SCGs (Q. A. Nguyen, Cho, Trinh, Jeong, & Bae, 2017), bio-oils due to the high fatty acid content of coffee cherries (Vardon et al., 2013), bioactive compounds (Panusa, Zuorro, Lavecchia, Marrosu, & Petrucci, 2013), bio-polymers like Polyhydroxyalkanoates (PHAs) (Obruca, Benesova, Kucera, Petrik, & Marova, 2015) can be produced from SCGs. Recently, the utilization of SCGs in bio-product and chemical formation has been reviewed by Ahmed, Abolore, Jaiswal, and Jaiswal (2024), which may significantly impact the circular economy.

## 5 Innovative techniques for applications of Agri-food waste

Every year, agriculture and food processing industries generate substantial organic residues and related effluents. At the same time, majority of these by-products are either used as animal feed or burned for disposal. However, such residues are often rich in sugars, minerals, proteins etc. and should not be considered “waste” but rather as valuable raw material for other industrial processes (Mussatto et al., 2012). Table 2 depicts the chemical composition of diverse agri-food waste analyzed by different researchers. Similarly, a portion of agri-food biomass from the above-mentioned industrial categories can be recovered with the right approach. The residual biomass can be a carbon substrate in bioreactors to transform waste materials into high-value bio-based products and/or biofuels, such as sweeteners, pigments, biopolymers, and platform chemicals.

**Table 2:** Chemical composition of various Agri-Food Waste.

**S. No.**	**Agri-food waste**	**Chemical Composition (% *w*/w)**						**Reference**
		**Cellulose**	**Hemicellulose**	**Lignin**	**Ash (%)**	**Total Solids (%)**	**Moisture (%)**	
1	Sugarcane bagasse	30.2	56.7	13.4	1.9	91.6	4.8	El-Tayeb, Abdelhafez, Ali, & Ramadan, 2012
2	Rice straw	39.2	23.5	36.1	12.4	98.62	6.58	
3	Corn stalks	61.2	19.3	6.9	10.8	97.78	6.40	
4	Sawdust	45.1	28.1	24.2	1.2	98.54	1.12	
5	Sugar beet waste	26.3	18.5	2.5	4.8	87.5	12.4	
6	Barley straw	33.8	21.9	13.8	11	–	–	Nigam, Gupta, & Anthwal, 2009
7	Cotton stalks	58.5	14.4	21.5	9.98	–	7.45	
8	Oat straw	39.4	27.1	17.5	8	–	–	Martin et al., 2012
9	Soya stalks	34.5	24.8	19.8	10.39	**–**	11.84	Motte et al., 2013
10	Sunflower stalks	42.1	29.7	13.4	11.17	–	–	
11	Wheat straw	33	24.0	8.9	6.7	96	7	[Bibr bb0500][Bibr bb0550]
12	Potato peel waste	2.2	–	–	7.7	–	9.89	Al-Weshahy & Rao, 2012
13	Orange peel	9.21	10.5	0.84	3.5	–	11.86	Rivas, Torrado, Torre, Converti, & Domínguez, 2008
14	Pineapple peel	18.11	–	1.37		93.6	91	Paepatung et al., 2009

**Fig. 3:**
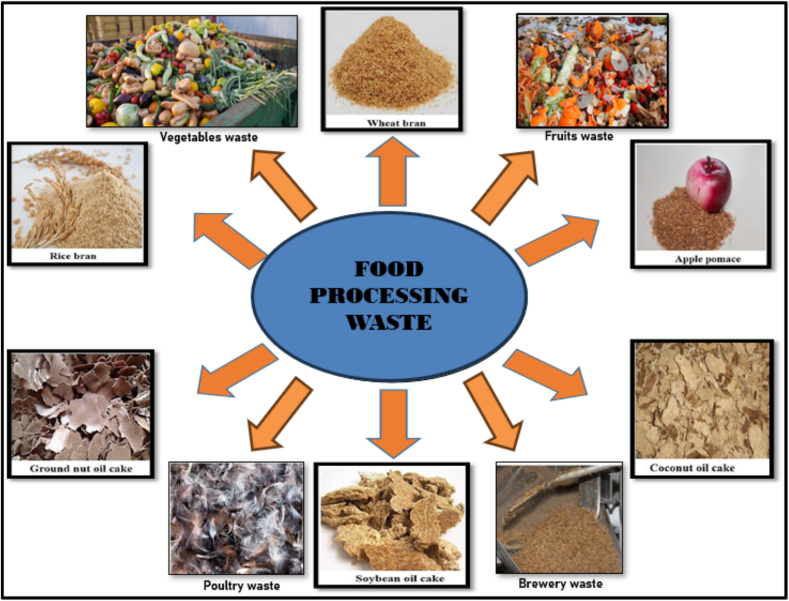
Examples of Agri-Food Waste that can act as Substrate for Value-Addition.

Agri-food waste has immense potential for transformation into valuable products through innovative valorization techniques. Fig. 3 depicts some of the wastes generated from food processing industries which can act as a considerable substrate for the production of value-added products. The diverse compositions of agriculture and food industry wastes, including cellulose, hemicellulose, lignin, ash, nitrogen, and other constituents, have the capability to be biochemically processed to generate valuable products such as biogas, bioethanol, and other commercially valuable items. Cereal residues, extracted grains, vegetable leftovers, and fruit waste (seed, skin, rind, pomace) are the major category of agricultural waste, which is a good source of potential bioactive components, like vitamins, proteins, minerals, carotenoids, polyphenols, dietary fibers, sugars, enzymes, and oils with high nutritional value and biological activity (Siles-Castellano et al., 2020; P. Kumar, Kumar, Kumar, Singh, & Kumar, 2020).

In India, approximately 20 % of the fruits and vegetables produced annually are wasted, mainly due to the country's substantial production of horticulture crops (Nirmal et al., 2023). Developing countries face the challenge of increasing agricultural production while minimizing the environmental impact of waste generated during processing. Therefore, there is an urgent need for technological innovations in the agriculture sector. Integrating biological sciences with technology has paved the way for finding promising solutions in food and agriculture, leading to innovative applications. Several novel techniques for agri-waste management have been fabricated, which help in reducing the toxic and hazardous effects of waste by converting it into eco-friendly value-added products. These conversions offer the potential of sustainable raw materials that can be utilized by living organisms, including humans (Akhtar et al., 2021). Furthermore, the extraction of bioactive components and their use in nutraceuticals and functional foods has been well documented to promote the health benefits (Rațu et al., 2023). Table 3 highlights the value-added applications of agri-food waste residues which are utilized for the production of enzymes, colors, flavors, organic acids, bioactive compounds, biodegradable polymers, biofuels etc.

**Table 3:** Value-added Applications of Agri-food Wastes Toward a Zero-Waste Circular Economy.

**S No.**	**Type of waste**	**Application**	**Reference**
**A**	**ENZYME PRODUCTION**		
1.	Mango kernel & cassava waste	Amylase	Kumar, Yadav, Muthukumar, & Garg, 2013 Pothiraj, Balaji, & Eyini, 2006
2.	Palm oil fiber & Banana waste	Cellulase; hemicellulase	Dabhi, Vyas, & Shelat, 2014 Xin & Geng, 2010
3.	Sapota peel & wastes from pineapple, banana, grapes and cashew apple	Pectinase	Venkatesh, Pushpalatha, Sheela, & Girija, 2009 Sabika Akbar & Prasuna, 2012
4.	Grape peel	Tannase	Paranthaman, Vidyalakshmi, Murugesh, & Singaravadivel, 2009
5.	Sugarcane bagasse; Corn cobs; Candelilla stalks; Coconut husks	Elagitannase	Buenrostro-Figueroa et al., 2014

**B.**	**EXTRACTION OF BIOACTIVE COMPOUNDS**		
1.	Apple pomace	Pectin	Wang et al., 2020
		Phenolic acids (Chlorogenic acid, Caffeic acid, Ferulic acid)	Barreira, Arraibi, & Ferreira, 2019 Lavelli & Corti, 2011
		Flavonoids (Isorhamnetin, Kaempferol, Quercetin)	
2.	Beetroot pomace	Phenolic acids (Ferulic acid, Vanillic acid Caffeic acid, Protocatechuic acid)	Vulić et al., 2014
		Flavonoids (Catechin epicatechin, rutin)	
3.	Banana Peel	Phenolic acids (Ferulic acid, p-Coumaric acid, Caffeic acid, Sinapic acids)	Vu, Scarlett, & Vuong, 2018
		Flavolnols (Rutin, Quercetin, Kaempferol, Myricitin, Laricitrin)	
		Catechin, Epicatechin	
4.	Citrus fruits: Peel and pulp	Phenolic acids (Hydroxybenzoic acid, Caffeic acid); Flavones (Apigenin-glucoside, Diosmetin-glucoside); Flavanones (Eriocitrin, Hesperidin, Narirutin)	Mahato, Sharma, Sinha, & Cho, 2018 Russo et al., 2014 & Russo, Bonaccorsi, Inferrera, Dugo, & Mondello, 2015
5.	Citrus fruits: Seeds	Limonoids (Limonin, Nomilin, Obacunone, Ichangin)	Liu et al., 2019

**C.**	**ORGANIC ACIDS PRODUCTION**		
1.	Pineapple peel	Acetic acid	Raji, Jibril, Misau, & Danjuma, 2012
2.	Pineapple pulp waste & Banana peel	Citric acid	Kareem & Rahman, 2013 Bezalwar, Gomashe, Sanap, & Gulhane, 2013
3.	Fibrous residue of cassava & peels of orange, mango, sweet corn, potato,	Lactic acid	Jawad, Alkarkhi, Jason, Easa, & Norulaini, 2013 Mudaliyar, Sharma, & Kulkarni, 2012

**D.**	**PRODUCTION OF COLORS & FLAVORS**		
1.	Apple pomace & Mung bean waste flour	Carotenoids	Joshi, 2005
2.	Apple pomace	Torulene	Buzzini, 2001
3.	Orange peel	α-Terpineol	Badee, Helmy, & Morsy, 2011
4.	Coffee husk	Vanillin	Vaithanomsat & Apiwatanapiwat, 2009

**E.**	**PRODUCTION OF BIOFUELS**		
1.	Cassava plant residue; whey	Bioethanol	Zhang et al., 2013
2.	Vegetable waste		Mushimiyimana & Tallapragada, 2016
3.	Lignocellulosic agri-waste		Saini, Saini, & Tewari, 2015
4.	Tomato processing waste; vegetable waste	Bio-methanol	Thauer & Shima, 2006 Roati et al., 2012
5.	Effluents of dairy, winery and brewery	Biohydrogen	Yokoi et al., 2001 Kapdan & Kargi, 2006

**F.**	**BIODEGRADABLE POLYMERIC SYSTEM PRODUCTION**		
	Potato peels	Chitosen blended biodegradable membranes	Taha et al., 2024
	Blueberry residue	Corn starch blended pH-indicator films	Luchese, Sperotto, Spada, & Tessaro, 2017

**H.**	**SINGLE CELL PROTEIN (SCP) PRODUCTION**		
1.	Peels of cucumber and orange	Single Cell Protein	Mondal, Sengupta, Bhowal, & Bhattacharya, 2012
2.	Peels of pineapple, orange, potato, banana and carrot pulp		Khan, Asif, Razzaq, Nazir, & Maan, 2022 Dias, Sajiwanie, & Rathnayaka, 2020
3.	Pineapple waste		Mensah & Twumasi, 2017
4.	Orange, pineapple, banana, watermelon and cucumber waste		Oshoma & Eguakun-Owie, 2018

### 5.1 As a substrate for solid-state fermentation

SSF is a biotechnological process in which organisms grow on non-soluble materials or solid substrates, typically in near or complete absence of free water (Bhargav, Panda, Ali, & Javed, 2008). Cereal grains (such as rice, wheat, barley, and corn), legume seeds, wheat bran, lignocellulose materials like straws, sawdust, or wood shavings, as well as various plant and animal materials are commonly used in SSF. The compounds in these substrates either remain insoluble or sparingly soluble in water and are polymeric in nature. However, most of them are inexpensive, readily accessible, and a rich source of nutrients for microbial growth. Moreover, using agricultural waste in fermentation is an easy and advantageous choice for waste recyclizing.

Carbon sources, nutrients, and moisture present in various agri-food wastes create favourable conditions for the growth of microorganisms which offers significant potential for their reuse in SSF processes. For instance, agro-industrial waste can serve as a solid support and a carbon/nutrient source in SSF processes to produce a range of value-added compounds. The lignocellulose content from the cereal (maize, wheat, rice) residue and leaves (banana and pineapple) can be utilized for the industrial production of high-quality, natural cellulose fibers (Babu et al., 2022). Lactic acid production using agri-waste as a substrate is another cost-effective and environmentally friendly approach, as lactic acid has a wide range of industrial applications in different sectors (Lima, Soares, & Martin, 2023). The valorization of agri-waste biomass for producing single cell proteins (SCP) as a value-added byproduct possesses multiple benefits like low cost, high nutritional value, non-toxicity, and usability as feed (S. Singh et al., 2023).

Different studies have explored the use of rice (*Oryza sativa*) (Sadh, Saharan, Duhan and Duhan, 2017), seim (*Lablab purpureus*) (Sadh, Saharan, & Duhan, 2017), black-eyed pea (*Vignaunguiculata*) (Chawla et al., 2017), and peanut press cake (*Arachis hypogea*) for SSF (Sadh, Chawla, Bhandari, Kaushik, & Duhan, 2017). Orzua et al. (2009) investigated ten types of agri-industrial wastes to assess their potential as carriers for fungus immobilization in SSF (Orzua et al., 2009). The results demonstrated that certain waste materials showed highly promising results as immobilization carriers due to their high water-absorption capacity and ability to support good microbial growth (Sadh, Duhan, & Duhan, 2018). Armada, Portela, Roldán, and Azcón (2014) observed an improvement in rock phosphate solubilization using citric acid derived from *Aspergillus* Sp. through SSF on agri-food waste (e.g. sugarcane bagasse).

The role of SSF for the production of several antibiotics from agri-food waste has been well recognized. Among various agri-industrial wastes, coconut oil cake and groundnut shells have been extensively studied for the production of antibiotics. The researchers have manufactured oxytetracycline using *Streptomyces* strains through SSF from groundnut shells (Asagbra, Sanni, & Oyewole, 2005) and household kitchen waste (Singh, Solanki, et al., 2020)*.* An antibiotic named lovastatin was produced from groundnut shell using *A. terreus* in SSF under optimum conditions of temperature (30 °C) and pH (5.5) with 10 days of fermentation period (Singh, Yousuf, & Mahapatra, 2020). Other than this, the production of tetracycline was reported using various strains of *Streptomyces* and pineapple peels as a potential substrate for SSF (Vastrad & Neelagund, 2011). The utilization of low-cost carbon sources from multiple agricultural residues has been stated to lower the antibiotic production costs significantly (Ul-Islam, Ullah, Khan, & Park, 2020). These residues hold great potential as an alternative for producing neomycin and other antibiotics.

### 5.2 As a substrate for biofuel production

Biofuels always remain a potential alternative to fossil fuels. Several studies have discovered biofuel production from agri-food residues like rice straw, sawdust, sweet potato, potato and sugar beet waste, corn stalks, and sugarcane bagasse (Hiloidhari et al., 2020; Singh et al., 2021). An eco-friendly approach to bioethanol production, considering the lignocellulosic content of agro-industrial residues offers a sustainable energy alternative (Sharma & Saini, 2020). Two weeds viz. *Typha angustifolia*, *Eichhornia crassipes*, and agri residue from different sources were investigated for biogas production (Paepatung, Nopharatana, & Songkasiri, 2009). Bioethanol production from kitchen leftovers (like, potato, carrot, and onion peels) by fermentation method using *S. cerevisiae* was also achieved (Mushimiyimana & Tallapragada, 2016) which can act as an efficient and cost-effective energy source in developing countries. In India, banana pseudo-stem waste is abundant, making it a potential substrate for bioethanol production. Ingale, Joshi, and Gupte (2014) used banana pseudo-stem as a substrate, pre-treated it with *A. ellipticus* and *A. fumigatus* to enhance cellulolytic activity and reported the production of bioethanol. Maiti et al. (2016) used starch industry wastewater (SIW) as agri-food waste to produce bio-butanol by using *Clostridium beijerinckii* and achieved a maximum yield of 11.04 g/L after 96 h of fermentation. The production of bioethanol/biofuel and the process productivity depends upon the chemical composition of raw material as well as the technique used for the production (Dutra Fagundes, Machado, R. De C., & Colla, 2025). Apart from this, several other process parameters such as pH, temperature, oxygen supply, type of substrate and microbial concentration are significantly influencing the production of bio-ethanol with minimum by-product (like, acetic acid) (Woźniak, Kuligowski, Świerczek, & Cenian, 2025). The hydrolysis treatment with different acids such as H_2_SO_4_, H_3_PO_4_, and HNO_3_ largly affects the decomposition of waste materials and the release of fermentable glucose (Osmolak, Mikulski, & Kłosowski, 2025). This study showed that yeast had the highest fermentation activity in the presence of stillage biomass as well as molasses in the media and bio-ethanol production of about 50 g/l was achieved, regardless the applied fermentation strategy (Osmolak et al., 2025). In another study, the production of bio-ethanol from waste potato mash increased to a concentration level of 61.8 g/l on using ultrasonic-assisted acid/enzymatic hydrolysis as compared to the conventional acid/enzymatic hydrolysis which produced 47.15 g/l of bio-ethanol. This increase in bio-ethanol production was due to the application of ultrasonic assisted acid/enzymatic hydrolysis which improved the yield of total reducing sugars by 29.17 %. This demonstrates that optimization of process parameters and adoption of improved technology could help in achieving the higher process efficiency (R. Kumar, Mondal, Sadhukhan, & Ganguly, 2025). Moreover, the use of low-cost, sustainable agricultural waste for biofuel production could be one of the promising ways to meet the energy needs through limited resources.

## 6 Opportunities and challenges in Agri-food waste valorisation

Agri-food waste is a rich source of valuable bioactive compounds such as dietary fibers, polyphenols, carotenoids, essential oils etc. All these components have potential applications in industries like food, pharmaceuticals, cosmetics and energy generation (Yadav et al., 2024). For instance, citrus peels and tomato waste contain phytochemicals with antioxidant and antimicrobial properties, which can address global health issues like antimicrobial resistance (Pereira, Berenguer, & Câmara, 2023; Roy, Mohanty, Dick, & Misra, 2023). Valorisation aligns with circular economy principles by turning waste into value-added products, reducing landfill use, and mitigating greenhouse gas emissions (Roy et al., 2023). Sustainable extractions methods like, ultrasound-assisted extraction, supercritical fluid extraction, are being fabricated for the efficient recovery of bioactive compounds. Further, agri-food waste valorisation can create added sources of income for industries by producing high-value products like, functional foods, biofuels, compost, and bioplastics (Pereira et al., 2023; Roy et al., 2023; Yadav et al., 2024).

Nevertheless, the high cost of advanced technologies (e.g., enzyme production for fermentation) limits the process of scalability. Additionally, the processes optimization for diverse types of agri-food waste remains challenging (Roy et al., 2023). Several factors viz., availability of feedstock, cost of processing, market demand for end products plays a crucial role in deciding the cost-effectiveness of valorization processes. Also, some valorization techniques (e.g., incineration) may lead to environmental burdens such as greenhouse gas emissions, if not managed properly. The scarcity of clear guidelines/regulations for agri-food waste management may obstruct the adoption of valorization practices. The policies promoting zero-waste strategies are still evolving in many regions. Nonetheless, it is challenging to ensure the consistent quality and safety of the products derived from agri-food waste due to variability in raw materials. Effective valorization requires coordinated efforts among farmers, industries, policymakers, and researchers to develop integrated solutions.

## 7 Possible solutions and future recommendations for Agri-food waste management

Our study suggests that the practices such as composting, anaerobic digestion, and upcycling food waste into biofuels or biofertilizers should be prioritized to enhance sustainability and resource efficiency. Most of the studies have described the successful up-cycling of agri-food waste, however more emphasis must be given on investment, research and policymaking to improve the necessary infrastructure for large scale valorization of agri-food waste. Moreover, Agri-food waste management can be done by adopting circular economy principles, reusing and recycling materials, converting waste into valuable products, and minimizing resource consumption throughout the food supply chain. Encouraging research and development in biotechnology can lead to innovative solutions for managing agri-food waste. To support a sustainable and circular economy, modern technologies such as bioconversion processes that transform organic waste into renewable energy sources (e.g., biogas) or biodegradable packaging materials can help mitigate waste generation while providing sustainable alternatives. Urban areas have abundant components such as peels, pomace, and seeds in their food waste from fruit and vegetable processing enterprises. By recovering high-value components from agri-industrial wastes, such as proteins, fibers, polysaccharides, phytochemicals, and flavor compounds, utilized as functional ingredients, valuable goods can be furnished. In addition to their use in cosmetics, pharmaceuticals, and nutraceuticals, such discoveries and value-addition can help us in achieving the SDGs. Hence, novel technologies should be explored in near future, for more sustainable solutions of waste management in agri-food industries. Raising awareness about food waste management within local communities is crucial. Educational programs can empower consumers and producers to adopt better practices, such as proper storage techniques and reducing food waste at home. Initiatives like “Food Rescue” systems can facilitate the redistribution of surplus food to those in need, fostering a sense of community responsibility.

## 8 Conclusion

Agri-food waste is one of the most abundant biomasses generated globally in the form of by-products during processing of fruits, vegetables, cereals, pulses, oil seeds, meat, fish, poultry, dairy, etc. Agricultural waste can be solids, liquids, or slurries, depending on the type of farming activities. More than 30 % of fresh agri-food produce is lost at different points during processing, leading to serious environmental and human health risks, when disposed of openly. The currently implied conventional practices like landfilling and incineration lead to massive economic loss. The valorization of agri-food waste could be a promising opportunity for sustainable development, environmental protection, and economic growth. The development of cost-effective and scalable biotechnological approaches can enhance commercial viability of waste valorization by converting it into valuable products like biofuels, bioplastics, organic fertilizers, and functional food ingredients. Anaerobic digestion, composting, solid state fermentation of agri-food waste as well as extraction of valuable bioactive compounds have been discovered as some promising methods for valorization. However, more emphasis must be given on encouraging research and development in waste-to-resource conversion technologies, development of sustainable biodegradable products- particularly packaging materials- and raising awareness among both processors and consumers for a more sustainable future. Additionally, agri-food processing industries must be motivated to adopt practices for reutilizing agri-food waste which could be a crucial step toward effective waste management and in advancing the principles of circular economy.

## CRediT authorship contribution statement

**Arun Kumar Pandey:** Writing – original draft, Investigation, Conceptualization. **Sheetal Thakur:** Writing – original draft, Methodology, Formal analysis, Conceptualization. **Rahul Mehra:** Writing – original draft, Visualization, Data curation, Conceptualization. **Raj Sukhwinder Singh Kaler:** Validation, Formal analysis. **Maman Paul:** Validation, Formal analysis. **Arun Kumar:** Validation, Funding acquisition, Formal analysis.

## Ethical approval

Ethics approval was not required for this study. The experiments were not conducted on humans or animals.

## Funding Information

AK acknowledges the financial support from Graphic Era (Deemed to be University), Dehradun, Uttarakhand India.

## Declaration of competing interest

The authors declare that they have no known competing financial interests or personal relationships that could have appeared to influence the work reported in this paper.

## Data Availability

The data that support the findings of this study are available from the corresponding author upon reasonable request.
